# Follicular helper T cell in immunity and autoimmunity

**DOI:** 10.1590/1414-431X20165209

**Published:** 2016-04-19

**Authors:** D. Mesquita, W.M. Cruvinel, L.S. Resende, F.V. Mesquita, N.P. Silva, N.O.S. Câmara, L.E.C. Andrade

**Affiliations:** 1 Divisão de Reumatologia, Escola Paulista de Medicina, Universidade Federal de São Paulo, São Paulo, SP, Brasil; 2 Divisão de Farmacologia, Instituto de Ciências Biológicas, Universidade de São Paulo, São Paulo, SP, Brasil; 3 Divisão de Imunologia, Faculdade de Medicina, Universidade de São Paulo, São Paulo, SP, Brasil; 4 Escola de Ciências Médicas, Farmacêuticas e Biomédicas, Pontifícia Universidade Católica de Goiás, Goiânia, GO, Brasil

**Keywords:** Autoimmune diseases, Tfh, Lymphoid tissue, Humoral immunity

## Abstract

The traditional concept that effector T helper (Th) responses are mediated by Th1/Th2 cell subtypes has been broadened by the recent demonstration of two new effector T helper cells, the IL-17 producing cells (Th17) and the follicular helper T cells (Tfh). These new subsets have many features in common, such as the ability to produce IL-21 and to express the IL-23 receptor (IL23R), the inducible co-stimulatory molecule ICOS, and the transcription factor c-Maf, all of them essential for expansion and establishment of the final pool of both subsets. Tfh cells differ from Th17 by their ability to home to B cell areas in secondary lymphoid tissue through interactions mediated by the chemokine receptor CXCR5 and its ligand CXCL13. These CXCR5^+^ CD4^+^ T cells are considered an effector T cell type specialized in B cell help, with a transcriptional profile distinct from Th1 and Th2 cells. The role of Tfh cells and its primary product, IL-21, on B-cell activation and differentiation is essential for humoral immunity against infectious agents. However, when deregulated, Tfh cells could represent an important mechanism contributing to exacerbated humoral response and autoantibody production in autoimmune diseases. This review highlights the importance of Tfh cells by focusing on their biology and differentiation processes in the context of normal immune response to infectious microorganisms and their role in the pathogenesis of autoimmune diseases.

## Introduction

The production of high-affinity class-switched antibodies is necessary for the clearance of pathogens after infection, for the establishment of long-term humoral immunity and for the effectiveness of vaccines (1). Follicular helper T (Tfh) cells have been recently shown to play a crucial role in instructing B cells to form a repertoire of antibody producing cells that provide life-long supply of high affinity, pathogen-specific antibodies (2).

**Figure 1:**
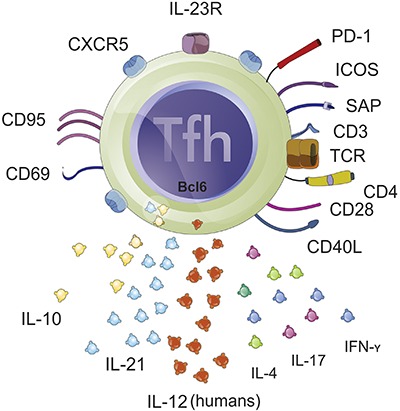
Follicular helper T cells (Tfh) lymphocytes are critically involved in the formation of germinal centers and in the development of T cell-dependent B cell response in secondary lymphoid tissues. The figure represents Tfh cells with their most important membrane molecules, transcription factor and soluble effector molecules.

Adaptive immune responses are regulated by the fine-tuning of the functional activity of several T cell subsets through a complex mechanism that integrates signals from innate immune cells and the cytokine milieu acting over naive T and B cells. Several CD4^+^ T cell subsets functionally distinct from the traditional Th1 and Th2 subsets have been described, displaying either effector (Tfh, Th17 and Th9) or regulatory functions (natural and inducible regulatory T cells, Tr1 and Th3) (3). It is a consensus that, during immune responses, many cell subsets are directly involved as effector cells in the inflammatory process. Tfh cells are described as non-polarized CD4^+^ T cells that express the highest levels of the chemokine receptor CXCR5, which is critical for their homing and function. Other distinguishing features of Tfh cells include the expression of the surface receptors inducible T cell co-stimulator (ICOS) and programmed cell death protein 1 (PD1; also known as PDCD1) as well as the nuclear transcriptional repressor B cell lymphoma 6 (bcl-6) (Figure 1). Tfh cells express high levels of IL-21 and other cytokines that influence B cell differentiation and antibody production. Also, they have down-regulated the T cell zone-homing receptor CC-chemokine receptor 7 (CCR7) and IL-7 receptor-α (IL-7Rα) (4). Due to this profile of receptors and cytokines, Tfh cells have the unique ability to home to B cell follicles and to induce antibody production during co-culture with B cells (5).

The B cell maturation process requires cognate help provided by CD4^+^ T cells in the T cell-rich extra follicular areas of secondary lymphoid organs. These are structures within the B cell follicles of lymphoid organs that support intense B cell proliferation and differentiation, somatic hypermutation, selection of high-affinity B cells, and class switching of immunoglobulin genes. B cells ultimately are differentiated into memory B cells and long-lived plasma cells that secrete high-affinity antibodies (6). In fact, without Tfh cells, germinal center do not develop, long-lived plasma cells are not generated, and long-term antibody responses are impaired (7). Memory B cells mediate long-term protective immunity due to their capacity to generate secondary humoral responses that surpass naive B cell responses by rapidly differentiating into high-affinity plasma cells (PC). Of interest, novel B cell receptor specificities, including autoreactive ones, arise continuously during the ongoing processes inside germinal centers. Antibody secreting cells that emerge from the germinal centers need to be tightly controlled due to their longevity and high-affinity antibody production. Autoantibody production indicates a profound breakdown in humoral tolerance mechanisms and B cell hyperactivity caused by either B cell-intrinsic abnormalities or immune-regulatory defects in other cell types.

In the last few years, significant progress has been made in the study of Tfh cells and there has been a surge of research activity aimed at understanding the function and differentiation of these important cells (1), examining the biochemistry of transcription factors involved in programming Tfh cell differentiation, and exploring the cellular biology of Tfh cell-mediated selection of germinal center B cells (8). Given the importance of Tfh in practically all T cell-dependent humoral responses, this review will address the most important biological aspects of this "new" lymphocyte subset in normal immune responses against infectious agents, and discuss its relevance on deregulated autoimmune processes.

## Tfh: An overview

In the early 2000's, a number of prominent studies in mice and in humans led to the identification of B follicular helper T cells, a subset of CD4 T cells localized in the tonsils and characterized by high expression of the master regulator bcl-6, which represses the expression of other T-cell subset-specific transcription factors and promotes the sustained expression of chemokine receptor CXCR5, which is essential for the migration of T cells into the B-cell follicular zones (5). Within the follicle, crosstalk occurs between B cells and Tfh cells, leading to class switch, recombination and affinity maturation (2).

**Figure 2:**
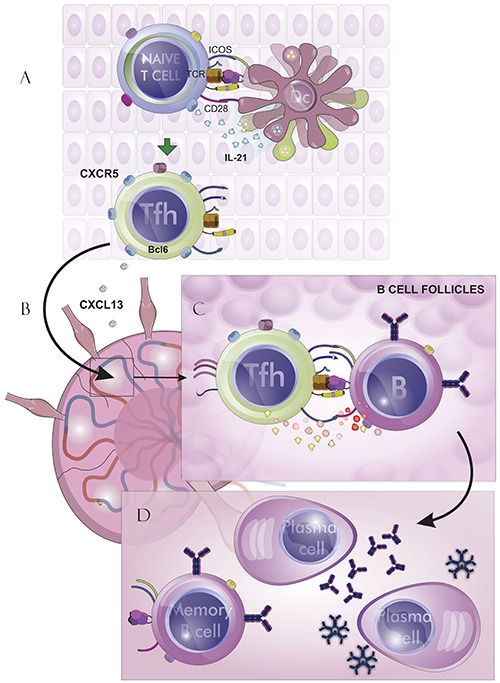
Schematic view of dentritic cell (DC) participation in the naive CD4^+^ T cells differentiation into follicular helper T cells (Tfh) lineage by means of an increase in Bcl-6 expression and downregulation of its antagonist Blimp-1 under the influence of a combination of elements, including IL-21, inducible co-stimulator (ICOS), T cell receptor (TCR) and likely CD28 (*A*). Since T cells are primed during interaction with DC in the T cell zone and B cells reside in the B cell follicle, antigen-specific T cells and their cognate B cells must migrate towards a secondary lymphoid organ to meet each other and promote the generation of germinal centers by differentiation of primed B cells (*B*).

Tfh cell differentiation is a multistage, multifactorial process with significant heterogeneity involving a variety of cytokines, surface molecules and transcription factors (4,8). Following immunization or infection, a cohort of naive CD4 T cells in the T cell zone acquire characteristics of pre-Tfh cells after interacting with dendritic cells (DCs) (9). The transformation of CD4^+^ T cells into Tfh lineage seems to be determined early during T-DC interaction by means of an increase in bcl-6 expression and downregulation of its antagonist Blimp-1 under the influence of a combination of elements, including IL-6, IL-21(mice) or IL-12 (humans), IL-2, inducible co-stimulator (ICOS), T cell receptor (TCR) and likely CD28 (8,), as depicted in Figure 2A.

CD4^+^ T cells bearing high affinity TCRs seem to preferentially suffer pre-Tfh differentiation over other effector T helper cells (13). Fazilleau et al. (14) showed that after the transfer of CD4^+^T cells into immunized mice, those T cells harboring TCRs with the highest affinity to peptide-MHC class II complexes and the most limited TCR diversity were selected into the Tfh cell pool. CD28 and ICOS also have an important role in the induction of Tfh cells differentiation and germinal center formation; CD28 appears to play a major role in the early phase of Tfh generation while ICOS is critical for generation of pre-Tfh cells as well as in later stages for Tfh differentiation (12).

The ability of activated CD4^+^ T cells to undergo differentiation into Tfh or into polarized effector T cells is dictated by the balance of cytokines that stimulate or prevent Tfh differentiation. For example, IL-6 and IL-21 cooperate to induce Tfh cell formation by activating STAT3, which in turn promotes bcl-6 and CXCR5 expression. In contrast, IL-2 signaling avoids Tfh cell development via STAT5 activation (7).

Several studies over the last few years have provided insights into the roles of these cytokines in Tfh cell commitment. Using different animal models of viral infection, researchers found varying and transient degrees of impairment in Tfh cell numbers in the absence of IL-6. Choi et al. (15) found that early bcl-6(+) CXCR5(+) Tfh differentiation was severely suppressed in the absence of IL-6; however, STAT3 deficiency failed to recapitulate that defect. IL-6R signaling activates the transcription factor STAT1 specifically in CD4^+^ T cells. Furthermore, IL-6-mediated STAT3 activation is important for downregulation of IL-2Rα, which contributes to limit Th1 cell differentiation in an acute viral infection. Thus, IL-6 signaling is an early inducer of the Tfh differentiation program mediated by both STAT3 and STAT1 transcription factors (15). According with this, Karnowski et al. (16) found that IL-6 production in follicular B cells in the draining lymph node was an important early event during antiviral response and that B cell-derived IL-6 was necessary to induce IL-21 from CD4^+^ T cells *in vitro* and to support Tfh cell development *in vivo*.

The requirement of IL-21 for Tfh cell differentiation from naive T cells and formation of the germinal center has been consistently demonstrated by several groups (11,17,18). Furthermore, recent studies convincingly showed that IL-12 can also drive Tfh cell differentiation by inducing IL-21 in a STAT3-dependent manner both in mice and in humans (10,19). Interestingly, a recent study provided evidence for a pivotal role of IL-7 in Tfh generation and germinal center formation *in vivo*, as treatment with anti-IL-7 neutralizing antibody, which markedly impaired the development of Tfh cells and IgG responses. Moreover, co-delivery of mouse Fc-fused IL-7 (IL-7-mFc) with a vaccine enhanced the generation of germinal center B cells as well as Tfh cells but not of other lineages of T helper cells, including Th1, Th2, and Th17 cells (6).

IL-2 is considered a canonical growth factor for CD4^+^ and CD8^+^ T cells (7). IL-2 signaling probably disturbs the differentiation of Tfh by stimulating Blimp-1 expression via STAT5 or by inducing the expression of T-bet, which forms complexes with bcl-6 masking the DNA-binding domain of bcl-6 and preventing bcl-6 from repressing the expression of Blimp-1 (20). Early studies found that the transcription repressor bcl-6 is necessary (when ectopically overexpressed) for programming Tfh cells, including CXCR5 expression. CD4^+^ T cells from *bcl6* ^−/−^ mice are impaired in the production of CXCR5^+^ Tfh cells *in vivo* whereas the differentiation of other CD4^+^ T cell subsets is relatively unaffected by the loss of bcl-6. This transcription factor acts in part by repressing the transcription of Tbx21 [encoding T-box expressed in T cells (T-bet)] and Rorc [encoding retinoic acid-related orphan receptor γt (RORγt)] or by direct binding to GATA-bind protein 3 (GATA3) (11,18). However, a study conducted by Liu et al. (21), using bcl-6-RFP reporter mice and phenotypic, functional and genome-wide transcriptome analysis of Tfh cells generated *in vivo*, found that the initial up-regulation of CXCR5 was not dependent on bcl-6, but once bcl-6 is highly expressed, Tfh cells can persist *in vivo* and some of them develop into memory cells.

Recently, Liu et al. (22) showed that the expression of transcription factor achaete-scute homologue 2 (Ascl2) is selectively upregulated in Tfh cells. Ectopic expression of *Ascl2* upregulates CXCR5 but not bcl-6, and down regulates CCR7 expression in T cells *in vitro*, as well as accelerates T-cell migration to the follicles and Tfh cell development *in vivo* in mice. Furthermore, studies indicate that Ascl2 directly regulates Tfh-related genes and inhibits the expression of Th1 and Th17 signature genes. Deletion of Ascl2, as well as blockade of its function with the Id3 protein in CD4^+^ T cells, results in impaired Tfh cell development and germinal center response (22). In addition to bcl-6, Ascl-2 and STAT3, other transcription factors are also known to be crucial for Tfh cell development, such as the basic leucine zipper transcription factor (BATF) (23) and the IFN regulatory factor 4 (IRF4) (24). It is interesting to note that STAT3, BATF, and IRF4 are also needed for differentiation of the Th17 cell lineage.

Since T cells are primed during interaction with DC in the T cell zone and B cells reside in the B cell follicle, antigen-specific T cells and their cognate B cells must migrate towards a secondary lymphoid organ to meet each other. This process is required for the generation of germinal centers and the differentiation of primed B cells along both germinal centers and extra follicular pathways (Figure 2B).

Tfh cells have a high ability to stimulate naive B-lymphocytes present in the follicle germinal center of secondary lymphoid organs by engaging B cells through co-stimulator molecules like CD40L, ICOS and SAP, and by producing important cytokines to humoral response as IL-10 and IL-21. Tfh cells produce also a diversity of cytokines, such as INF-γ and IL-4, which direct B cells antibody isotype commitment (25), and IL-17, a pro-inflammatory cytokine, recently reported as an important B cell factor, directly influencing its survival, proliferation and differentiation (26). IL-4-producing Tfh cells induce B cell IgG1 switch, and IFN-γ-producing Tfh cells induce B cell IgG2a switch. Interestingly, high-affinity IgG1 antibodies could only be induced by IL-4 produced by Tfh cells (25).

A cluster of microRNAs (miRNAs) known as miR17-92 has been recently reported to have a regulatory role on Tfh cell differentiation and in germinal center reaction. Initially, bcl-6 was proposed to repress the miR17-92 inhibiting effect over Tfh cell development (18). However, more recent studies show that miR17-92 cluster acts as a positive regulator of Tfh cell differentiation since mice with T cell-specific deletion of miR17-92 cluster (tKO mice) exhibit severely compromised Tfh differentiation, germinal center formation and antibody responses (27).

The inducible co-stimulator (ICOS) is another highly expressed molecule in Tfh cells and is essential for both Tfh differentiation and its effector function over B cells. The importance of ICOS is highlighted by the multiple ways in which ICOS signaling is regulated. Roquin inhibits ICOS, and combined loss of Roquin 1 and Roquin 2 results in spontaneous Tfh cell and germinal center development (28). A study suggested that ICOS is also essential for Th17 cell development (29); however, it has been shown that its importance for these cells is mostly associated with cell survival and to its function by regulating IL-21 production, which contributes to the expression and maintenance of IL-23R. In addition to the dependency to ICOS, Tfh and Th17 cells have more features in common. Both subsets produce IL-21 and IL-17, express IL-23R and are dependent of the transcription factors c-Maf and Stat3 to expand and produce IL-21. However, Th17 cells express the transcription factor RORy that is neither expressed by Tfh cells nor necessary for its development. Tfh and Th17 cells also differ in the ability to home to different immune microenvironments; while most Tfh cells are CXCR5^+^ and migrate to the secondary lymphoid tissue B cell areas, Th17 cells, when activated, down regulate CCR7 and up regulate CCR6, migrating to the target organs where they exert their effector functions. However, one may not exclude the possibility that some Th17 cells, with high expression of ICOS and IL-23R, may down regulate CCR7 and up regulate CXCR5 becoming part of the heterogeneous Tfh cell pool that migrates to the follicles. When activated, Tfh cells also express other non-specific markers such as CD69, CD95 and CD40L, but none of them characterizes these cells as a distinct subset (29).

As mentioned before, it was recently shown that Tfh cells develop preferentially from naive T cells with high avidity TCR. In the same study the authors proposed the existence of three Tfh compartments based on their migratory properties and molecular characteristics (14). Evidence indicates that depending on the cytokine microenvironment and the nature of the APC-activating antigen, naive CD4^+^ T cells acquire specific chemokine receptors that dictate their migration to a specific environment and their fate as specific Th subset. In fact, recent studies in mice and humans show that Tfh lineage cells are composed of subsets that differ in their localization, phenotype and function. The compartment of circulating Tfh memory cells in human blood contains heterogeneous subsets that differ in phenotype and function (30). Growing evidence has demonstrated that dysfunction of Tfh cells results in abnormal positive selection of autoreactive B cells, which contributes to the development of autoimmune diseases.

## Tfh and autoimmune diseases

The avoidance of autoimmunity is heavily dependent of T and B lymphocyte tolerance mechanisms. It is postulated that progressive breakdown in the tolerance mechanism would involve an increasing variety of cells. The classical example is shown by murine models for studying CD4^+^ T cells tolerance, in which a single disturb in CD4 helper responses can result in both cellular and humoral mediated immune responses against self-antigens (31). This occurs because most B cell responses depend on T cell help. The absence of T cell help during B cell priming, a mechanism of peripheral tolerance, leads to apoptosis or anergy; B cells lose their access to lymphoid follicles and the chance to differentiate into germinal center cells or plasma cells. How auto-reactive B cells avoid central tolerance in bone marrow and how they evade peripheral tolerance to access follicles remains an area of active investigation. The exclusion of self-reactive B cells from germinal center has been shown to be defective in systemic lupus erythematosus (SLE) patients, and spontaneous germinal center organization has been observed in different murine models of lupus (32). Another possibility is that B cells become auto-reactive after gaining access to the follicle. In this situation, after foreign antigen recognition in germinal centers, B cells undergoing affinity maturation, which improves the receptor affinity by somatic mutations, accidentally would give rise to auto-reactive cells. In SLE patients and murine models of lupus, auto-reactive B cells become competent to produce autoantibodies, mostly high avidity IgG (33).

Whether and how Tfh cells collaborate to the pathogenesis of human autoimmunity is not clear. Recent advance in understanding the biology of peripheral memory Tfh cells has rendered the analysis of human Tfh responses in the context of autoimmunity feasible (30). Different groups have studied the presence of circulating Tfh cells as a potential biomarker of disease in various autoimmune conditions, including myasthenia gravis (MG), autoimmune thyroiditis, Sjögren’s syndrome (SS), rheumatoid arthritis (RA), multiple sclerosis (MS), systemic lupus erythematosus, ulcerative colitis, Crohn's disease, ankylosing spondylitis, type 1 diabetes mellitus (T1D), autoimmune hepatitis, primary biliary cirrhosis (Table 1) and juvenile dermatomyositis.

**Table:**
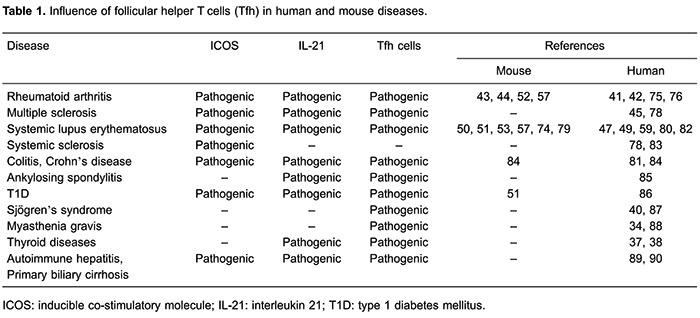

MG is an organ-specific autoimmune disease characterized by the T cell-dependent production of anti-acetylcholine receptor (AChR) antibodies. Patients with MG show a significantly higher frequency of CXCR5^+^ CD4^+^ T cells in the peripheral blood, which correlates with disease severity (34). Furthermore serum CXCL13 was found to be increased in MG patients and high CXCL13 serum level was associated with severe clinical stages (35). Interaction between CXCR5 and CXCL13 is especially required for B-cell architectural organization regulating compartmentalization of B- and T-cells in secondary lymphoid organs. Accordingly, Meraouna et al. (36) reported that CXCL13 expression was also increased in the thymus as well as in sera of MG patients not receiving glucocorticoid therapy and that CXCL13 level decreased with glucocorticoid treatment, in correlation with clinical improvement. Taken together, these results suggest dysregulation of blood CXCR5^+^ CD4^+^ T cells in MG patients and that serum CXCL13 reflects the general status of MG severity.

Also, in Graves' disease, the affected thyroid tissue showed a positive correlation of CXCR5 and CXCL13 mRNA expression with the number of lymphocytic infiltrates and ectopic germinal centers (37). Recent studies detected increased percentages of circulating Tfh cells in patients with autoimmune thyroid disease as well as a positive correlation between the percentages of circulating Tfh cells and the serum concentrations of antibodies against TSH receptor, thyroperoxidase and thyroglobulin (38).

Patients with juvenile dermatomyositis show a strong skewing of blood CXCR5^+^ Th cell subsets toward Th2 and Th17 phenotypes. Importantly, this skewing correlated with disease activity and the frequency of blood plasma blasts (39). In light of this observation, circulating CD4^+^ CXCR5^+^ T cells from patients with SS were recently re-examined with respect to the Th phenotypes. A positive correlation was found between the levels of serum autoantibodies and the numbers of circulating CD4^+^ CXCR5^+^ T cells, particularly with regard to those that also expressed CCR6. The frequency of Th17-like subsets (CD4^+^ CXCR5^+^ CCR6^+^) in SS patients was found to be significantly higher than in healthy controls. Functional assays showed that activated Th17-like subtypes in the peripheral blood display the key features of Tfh cells, including invariably co-expressed PD-1, ICOS, CD40L and IL-21. Th17 subsets were found to highly express bcl-6 protein in contrast to Th1 and Th2 cells that do not express these markers (40).

RA is another autoimmune disorder that has been recently studied with regard to Tfh cell dysregulation. Increased frequency of CD4^+^ CXCR5^+^ ICOS^high^ circulating Tfh cells was detected in the peripheral blood of RA patients, and this was positively correlated with high levels of serum anti-CCP antibody. Furthermore, increased expression of bcl-6 mRNA and plasma IL-21 concentrations was observed in these patients (41). Increased serum IL-21 levels in RA patients correlate with disease activity score, anti-CCP antibody titer and the frequency of circulating Tfh-like cells (42). In addition, Jang et al. (2009) reported that IL-21 receptor-deficient K/BxN mice have less severe RA with reduced Tfh cell population in draining lymph nodes (43). Platt et al. (44) found increased Tfh cells and antibody production in an OVA-induced RA mouse model.

The involvement of activated Tfh cells in MS was recently demonstrated by Christensen et al. (45). This study was the first to report prominent Tfh, Th17 and B-cell activation in the peripheral blood from patients with progressive MS, and these findings parallel recent pathology studies. Tfh and B cell activation correlated with disease progression and Tfh activation marker IL-21 was decreased in MS patients treated with mitoxantrone. Furthermore, there was increased expression of genes associated with Tfh and B cell activation in the cerebral-spinal fluid cells from MS patients. These findings emphasize an association between the systemic immune compartment and disease progression in the protected central nervous system environment.

Dysregulated activation of both T and B lymphocytes with overt production of auto-reactive antibodies is a hallmark of SLE. This prototypic systemic autoimmune disease is characterized by various immunologic abnormalities, including the presence of antibodies against double stranded DNA (dsDNA). Previous studies demonstrated that B cell chemokine CXCL13 is highly expressed in the thymus and kidneys in murine models for SLE (46). These results are in accordance with the study by Wong et al. (47), who showed that the significant increase in plasma concentration of CXCL13 in SLE patients correlated significantly with SLE disease severity. Recently, Le Coz et al. (48) found an increased proportion of Tfh cells in SLE patients with active disease. This increase was associated with key biological SLE parameters (total immunoglobulin serum levels and anti-dsDNA antibodies), B cell subset alterations and the presence of high IgE levels (49).

The relationship between Tfh cells and autoantibody production is evident in several mouse strains over or under expressing important Tfh cell-associated molecules such as ICOS, CD40L, SAP and IL-21 (50,51). In mice homozygous to Roquin gene mutation (Rc3h1 mice), both naive and activated T cells express abnormally high levels of ICOS, which apparently contributes to the development of SLE-like disease and early-onset diabetes (52). The disruption of ICOS-ICOSL signaling prevents autoantibody formation and organ inflammation in Rc3h1 mice and other murine models of autoimmune diseases, such as SLE-like disease, collagen-induced arthritis, and myasthenia gravis (53-56).

In addition, three murine lupus models (NZB\NZW, C57BL/6J (B6) and BXSB) and one arthritis model (collagen-induced arthritis) are known to be dependent on the maintenance of the ICOS pathway. They are characterized by a Tfh cell-like transcriptome, with excessive numbers of Tfh cells and germinal centers (51,57). Humans who are deficient in ICOS develop common variable immunodeficiency, in which there is impairment in the development of memory B cells and immunoglobulin class switch does not occur (58), highlighting the importance of the ICOS-ICOSL interaction for the development of an effective humoral response. It has been reported that ICOS signaling can stimulate Tfh cells to produce IL-10, which has been implicated (at high-levels) in the terminal differentiation of germinal center B cells into plasma cells (19). In this context, it is relevant that circulating CD4 and CD8 T cells from SLE and rheumatoid arthritis (RA) patients and synovial fluid cells from RA patients show increased ICOS expression (49,59).

However, Tfh cells are not the single effector T cell subtype that determine the dominant pathway of high-affinity isotype-switched autoantibody production in murine models of lupus. In the MRL/MpJ-Fas lpr murine model of lupus, a subset of CD162^low^CXCR4^+^ T cells, localized in extra-follicular sites, has been shown to mediate IgG production through IL-21 and CD40L. CD162^low^CXCR4^+^ T cells are abundant in other autoimmune murine models and can exhibit either a follicular or an extra-follicular phenotype (60). However, although isotype-switched autoantibody production may occur outside germinal centers, the process is far less efficient (48).

Similarly to ICOS, CD40L and SAP are essential for B cell effective help, and deficiency of either one results in a poor germinal-center humoral response with defective differentiation of both long-lived effector memory and plasma cells (61,62). In human patients and in mouse models of autoimmune diseases, an imbalanced expression of these molecules has been reported to contribute to the immune pathology, leading to maturation and differentiation of long-term antibody-secreting cells and autoantibody production (63). Some studies observed that the blockade of CD40L/CD40 interaction might be an efficient therapeutic approach to alleviate immunoglobulin secretion, autoantibody production and disease activity in lupus patients with proliferative glomerulonephritis (64,65). It was also reported that SAP deficiency prevents the development of experimentally induced SLE-like disease. An excessive Tfh number in Roquin^san/san^ (sanroque) mice is associated with spontaneous germinal center development, autoantibody production and lupus-like autoimmunity (66). In these mice, SAP deletion caused a substantial reduction in Tfh frequency, IL-21 levels, as well as reduced ICOS expression by Tfh cells. SAP deficiency also abrogated formation of germinal centers, autoantibody production and renal pathology in sanroque mice (67). Interestingly, the adoptive transfer of sanroque Tfh cells caused spontaneous germinal center formation in wild type mice. Altogether, these findings indicate a noticeable causative link between Tfh dysfunction and systemic autoimmune pathways (66).

## IL-21 implication

IL-21 has arisen as a powerful inducer of human B cell differentiation (68,69). It has been considered the most potent human T cell-derived cytokine for the induction of B cell proliferation (69). In mice, this cytokine is largely produced by Th2, Th17, NKT and Tfh CD4 cells, however, in humans the T cell types responsible for IL-21 production are less well characterized (70,71), and Tfh cells are still considered one of the most important sources (70).

In mouse models, Tfh cells are a central source of IL-21 in germinal centers of secondary lymphoid organs, providing cognate help to B cells in the germinal center dynamic microenvironment, acting specially on naive B cells to induce isotype switch to IgG and IgA (68). IL-21 also acts on B cells of cord blood and peripheral blood inducing plasma cell differentiation (69), whereas IL-10, a well-known mediator of human B cell differentiation, has the same effect on terminally differentiated B cells (72). The ability of IL-21 to induce differentiation of naive B cells into plasma cells suggests that IL-21 may have a major role in primary responses to antigens (73). One study with IL-21-transgenic mice and using hydrodynamic injection of IL-21 plasmid-based methodologies into wild-type mice showed that IL-21 induced apoptosis just in a subset of mature B cells, but increased the number of immature and post switch B cells (74). Thus, it appears that IL-21 differentially influences B cell fate depending on the signaling context and B cell differentiation stage. This would explain how IL-21 can be pro-apoptotic for B cells in some *in vitro* experiments and yet critical for Ag-specific immunoglobulin production *in vivo*. In transgenic mice, IL-21 overexpression promotes the differentiation of activated B cells into plasma cells and unexpectedly induces expression of both Blimp-1 and bcl-6, indicating mechanisms by which IL-21 can serve as a complex regulator of B cell maturation and terminal differentiation. BXSB-Yaa mice, which develop SLE-like disease, have an increased serum expression of IL-21, suggesting a possible role for IL-21 in the development of the autoimmune disease in this animal model (74). Accordingly, other animal studies have indicated that the production of autoantibodies and systemic autoimmunity is associated with elevated production of IL-21, Tfh dysfunction within germinal centers and aberrant positive selection of germinal center B cells (50,66).

In human autoimmune diseases, IL-21 appears to have the potential to exacerbate cellular processes that determine the course of autoimmune response. In patients with RA, IL-21R is overexpressed in the inflamed synovial membrane and in leukocytes from peripheral blood (PB) and synovial fluid (SF). In addition, PB and SF T cells from RA patients, when stimulated with IL-21 and anti-CD3 monoclonal antibody, secreted markedly higher levels of TNF-α and IFN-γ than those from controls, indicating that IL-21 enhances local T-cell activation and pro-inflammatory cytokine secretion (75). The blockage of IL-21R signaling pathway may have a therapeutic potential in RA patients. In fact, blockade of IL-21 and IL-15, cytokines belonging to the common γ-chain receptor family, is effective to inhibit the release of pro-inflammatory cytokines (TNF-α, IL-6 and IL-1β) in RA synovial cell cultures (76). On the other hand, another study showed that IL-21R expression by fibroblasts and macrophages in RA synovium did not correlate with the destruction of articular cartilage and bone (77).

IL-21R mRNA was up-regulated in keratinocytes and dermal fibroblasts in biopsy specimens from patients with systemic sclerosis (SSc; scleroderma). In addition, *in situ* hybridization and immunohistochemical analysis showed up-regulation of IL-21R in samples of epidermis from SSc patients (78).

Polymorphism of IL-21 gene has also been reported to be associated with SLE (79), however it is not known whether this polymorphism is functional. IL-21 implication in human SLE remains to be more extensively investigated since in several murine models of SLE, IL-21 has been either directly or indirectly shown to be a contributing factor to disease (51,53). Recently, Dolff et al. (80) published the first study demonstrating increased proportions of circulating IL-21+ T-cells in SLE patients. Elevated plasma IL-21 in SLE is probably a result of Tfh cell activity in the formation of germinal centers (17).

In both Crohn's disease (CD) and ulcerative colitis (UC), the major forms of inflammatory bowel diseases (IBD) in humans, high IL-21 production was related to the pathological process. High levels of IL-21 were observed in the inflamed colon of most patients with UC. In addition, IL-21 stimulated gut fibroblasts to secrete extracellular matrix degrading enzymes and was involved in recruiting T cells to the inflamed gut by inducing MIP-3 α production by epithelial cells. Altogether, these data denote that IL-21 is an important mediator of the chronic inflammatory response in CD and UC, and might be a potential therapeutic target in IBD (81 [Bibr B82] [Bibr B83] [Bibr B84] [Bibr B85] [Bibr B86] [Bibr B87] [Bibr B88] [Bibr B89] [Bibr B90]).

## Concluding remarks

*In vivo* and *in vitro* experiments present considerable evidence that Tfh cells interact with B cells and play a critical role in the formation of germinal centers and in the development of T cell-dependent B cell response in secondary lymphoid tissues. Exaggerated expansion of Tfh cells results in excessive germinal center reaction, self-reactive B cell proliferation, and excess long-lived plasma cells differentiation, as well as overproduction of high-affinity pathogenic autoantibodies. The pathological abundance of Tfh cells could provide a crucial help for the cognate self-reactive B cells survival and escape from the tolerance checkpoints at the germinal center. These observations suggest an important role for Tfh cells in human autoimmunity. Alteration of Tfh cells have been reported in patients with various autoimmune diseases, such as rheumatoid arthritis, systemic lupus erythematosus and autoimmune thyroid diseases, where Tfh cells are present at increased frequency and show positive correlation with serum autoantibody titer. Therefore, a better understanding of the biology and roles of Tfh cells is expected to contribute in designing tools to abrogate the inappropriate activity of these cells. The intervention by agents selectively targeting specific signature molecules of Tfh cells, such as ICOS and IL-21, may prove to be therapeutically effective.
